# Implementation of checklists in health care; learning from high-reliability organisations

**DOI:** 10.1186/1757-7241-19-53

**Published:** 2011-10-03

**Authors:** Øyvind Thomassen, Ansgar Espeland, Eirik Søfteland, Hans Morten Lossius, Jon Kenneth Heltne, Guttorm Brattebø

**Affiliations:** 1 Department of Anaesthesia & Intensive Care, Haukeland University Hospital, Bergen, Norway; 2 Department of Radiology, Haukeland University Hospital, Bergen, Norway; 3 Department of Research, Norwegian Air Ambulance Foundation, Drøbak, Norway; 4 Department of Surgical Sciences, University of Bergen, Bergen, Norway; 5 Department of Medical Sciences, University of Bergen, Bergen, Norway; 6 Department of Prehospital Medicine. Betanien University College, Bergen, Norway

## Abstract

**Background:**

Checklists are common in some medical fields, including surgery, intensive care and emergency medicine. They can be an effective tool to improve care processes and reduce mortality and morbidity. Despite the seemingly rapid acceptance and dissemination of the checklist, there are few studies describing the actual process of developing and implementing such tools in health care. The aim of this study is to explore the experiences from checklist development and implementation in a group of non-medical, high reliability organisations (HROs).

**Method:**

A qualitative study based on key informant interviews and field visits followed by a Delphi approach. Eight informants, each with 10-30 years of checklist experience, were recruited from six different HROs.

**Results:**

The interviews generated 84 assertions and recommendations for checklist implementation. To achieve checklist acceptance and compliance, there must be a predefined need for which a checklist is considered a well suited solution. The end-users ("sharp-end") are the key stakeholders throughout the development and implementation process. Proximity and ownership must be assured through a thorough and wise process. All informants underlined the importance of short, self-developed, and operationally-suited checklists. Simulation is a valuable and widely used method for training, revision, and validation.

**Conclusion:**

Checklists have been a cornerstone of safety management in HROs for nearly a century, and are becoming increasingly popular in medicine. Acceptance and compliance are crucial for checklist implementation in health care. Experiences from HROs may provide valuable input to checklist implementation in healthcare.

## Introduction

It has been said that while medicine used to be inefficient, simple, and safe it is now effective, highly complex, and dangerous [1]. Adverse events are documented to affect more than one in 25 hospital patients [2]. Failure to check equipment and lack of vigilance are examples of factors associated with adverse events [3,4]. Realising how prone we as humans are for short term memory loss, it is striking how many potentially dangerous medical procedures are based on perfect memory [5]. In this context, it is rather strange that checklists are not used more often in medicine.

Checklists are common in some medical fields, and can be an effective tool to improve care processes and reduce mortality and morbidity [6-11]. Checklists are also shown to be a useful tool in pre-hospital and emergency medicine [12,13]. However, the development and implementation of medical checklists share some of the same challenges as other quality improvement work in medicine, and can be a complex and resource-demanding exercise. Despite the seemingly rapid acceptance and dissemination of the WHO Safe Surgery Checklist, there are few studies describing the actual process of developing and implementing such tools in health care [14,15].

High reliability organisations (HROs) are often referred to in the medical literature because they perform hazardous and complex operations with an exceptionally low failure rate [16,17]. Examples of HRO are aviation and aerospace industry, nuclear power production, fire fighting, military operations, and engineering.

Checklists are commonly used in HROs as cognitive aids, freeing mental capacity for the operation itself. These organisations have decades of experience with checklist development and implementation. HROs also have a deep understanding of both the demanding conditions under which the "sharp-end" operates, and how the "blunt-end" and "sharp-end" interact (table 1) [18]. Aviation has often been referred to among the non-medical HROs. We believe that the extensive experience gained in aviation and other HROs are a valuable and under-utilized source for learning and improving health care safety. The aim of this study was to explore ideas and lessons learned from checklist development and implementation in a group of non-medical HROs.

**Table 1:** Characteristics of HROs

Preoccupation with the possibility of failure
Resistance to oversimplification

Sensitivity to "sharp-end" operations

Commitment to resilience and self-preservation

Deference to shifting locations of expertise

## Methods

The study was approved by the local Institutional Research Ethics Committee. A triangulation of methods was chosen. In order to gain in-depth and cultural understanding, a qualitative approach with key informant interviews and field visits were used [19]. The results from the interviews were further analysed, by health care workers, using a Delphi process.

### Participants

To obtain a range of views, eight informants were recruited from six different HROs based on the literature and the authors' personal knowledge (table 2) [20,21]. The organisations were formally asked to appoint the informants. The candidates then underwent a pilot interview to ensure that they had comprehensive experience in checklist development and "sharp-end" use of checklists in addition to a comprehensive cultural understanding of their own organisation. All the informants were males, with 10-30 years of experience in high-risk operations. As the organisations all had restrictions on sharing experiences or standing operating procedures with the public, the informants had to have an organisational standing giving them permission to disclose and discuss potentially sensitive information.

**Table 2:** Data on the key informants and their organisations

Type of HRO	Organisation	Key informant's organisational rank & positions	Personal experience with checklist use (years)
Nuclear power	Forsmark, Sweden	Reactor operator & security manager	15
		
		Safety engineer	25

Off-shore drilling	Statoil ASA, Norway	Safety manager	20
		
		Safety coordinator	12

Civil aviation	Norwegian Air Ambulance Service	Captain and flight safety officer	30

Navy	Submarine Training Centre, Norway	Commanding officer	10

Military special operations	Air Force Special Operation Unit, Norway	Commanding officer	23

Military aviation	National Search & Rescue Service, Norway	Captain and deputy executive officer	11

### Interviews and analysis

**Figure 1:**
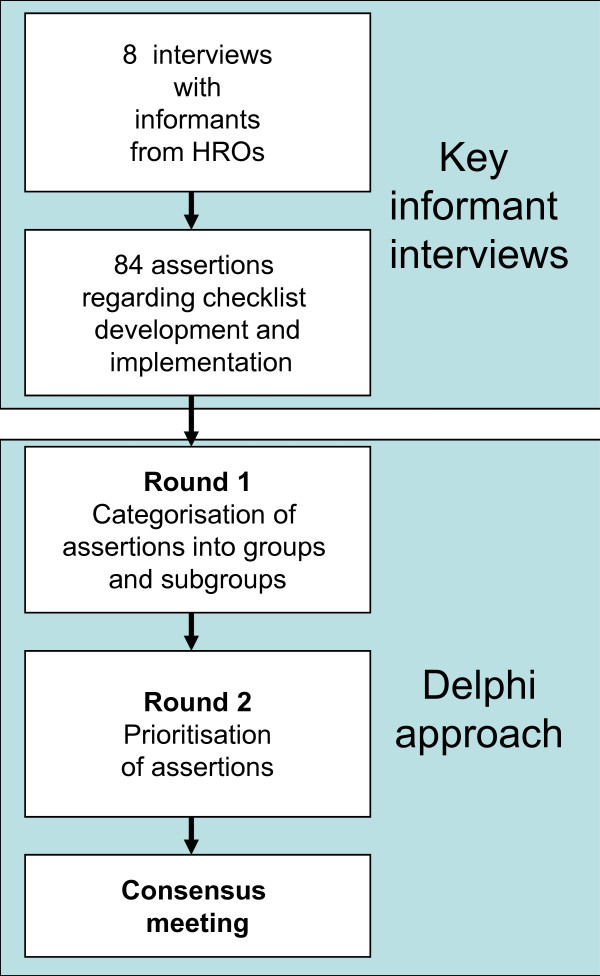
**Flow of the analysis of key informant interviews and application of the Delphi process**.

Six of the experts underwent a semi structured interview at their work place and two by telephone. The interview guide had broad, open-ended questions regarding the informants' personal experiences with checklists. The interviews (lasting 45 to 90 minutes) were tape recorded and transcribed verbatim. Furthermore, one of the authors (OT) made field visits to all the HROs to observe their checklist use and organisation. Field notes were taken during parts of interviews when audio recording was inconvenient or not permitted (e.g. during a tour of the nuclear plant or inside the parachute packing area). All transcripts and field notes were reviewed by two of the authors (OT & GB) who identified and agreed on 8-12 assertions from each informant regarding important issues and elements in checklist development and implementation. These assertions were returned to the informants for validation (member check), resulting in minor revisions. The authors (except AE), who all have previous experience with quality improvement projects, further analysed the assertions in a Delphi approach by e-mail and during a final consensus meeting [22,23]. In the first step of the process, each author proposed groups and subgroups of assertions. In the second step, the most important assertions were pinpointed. Finally, there was a consensus meeting where the groups and subgroups where further discussed. The lead authors (OT & GB) then performed the final analysis, and all authors agreed on the result. Figure 1 illustrates the entire process.

## Results

The key informant interviews generated 84 assertions and recommendations for checklist development and implementation (available from the corresponding author). Seventeen assertions were judged not to be transferrable to health care, and were excluded from further analysis. The assertions were categorized into five main groups with additional subgroups. Citations are in *italics *and the individual source is identified with capital letters.

### Assumptions for checklist acceptance

Several of the informants underlined that a checklist is a tool, not a goal in itself. In order to build a reliable safety culture and achieve checklist acceptance, two basic assumptions must be met; firstly, *"There must be a predefined problem that a checklist is the right tool for solving" *(C) and secondly, *"The end user must not get the feeling that he or she is deprived of the opportunity to apply common sense" *(A).

### Stakeholders in checklist development

All of the informants expressed that proximity to the checklist development process is very important. One informant cautioned about merely copying checklists; *"There must be a built-in scepticism as to whether the experiences of others are relevant for my organisation" *(B). Two informants said; *"Proximity between the checklist owner and the end user is important" *(D) and *"Checklists should be made within the organisation that intends to use it" *(G).

Within an organisation, there are many relevant parties. Table 3 lists some stakeholders involved in checklist development and implementation.

**Table 3:** Stakeholders in the checklist development and implementation process.

The "sharp-end" user	*"The end-user must be involved in the development process" *(F)
	
	*"The leader of the development process must be one of the 'sharp-end' users" *(D)
Head of department	*"The checklist must be developed by the users, but quality assured by the responsible head of the department" *(G)

Indirectly affected parties	*"If an operation for which a checklist is planned to be developed affects a third party, the third party must also be informed and consulted" *(H)

Well-recognised or leading person	*"Get an authority, a leading person, or someone with a good reputation to represent the checklist implementation" *(H)

### Characteristics of the checklist itself

#### Length

All informants agreed that limiting the length of a checklist is crucial for its feasibility and usefulness. One informant (E) referred to the resuscitation "ABC checklist". He believed that the reason that this checklist is well known worldwide is because of its simplicity (e.g. it follows the alphabet and is hierarchical, where A is more important than B, and B is more important than C). Another informant warned about the gradual extension of an initially short and well-adapted checklist; *"Be careful not to extend checklists because someone - often inexperienced personnel - calls for a more comprehensive list. Too many additions may result in a long and useless checklist" *(B).

#### Lay-out and design

*"Be extremely attentive to font and graphics" *(E). The informants had experiences with numerous design types. *"The design must be adapted to the surroundings, e.g. noise, light conditions, and vibrations" *(D). One informant recommended that a professional graphics designer be used to produce the final version of the checklist (G).

#### Content

According to the informants, none of the HROs used any specific validated methods for selecting the specific content of checklists, but some important issues from this process were mentioned. One informant (B) described the "20/80 rule" (the Pareto-principle). His organisation believed that 20% of the possible adverse events would cause 80% of the consequences. Hence, this principle was used to identify and prioritize the content of their checklists. Several informants emphasized the importance of a precise and operationally-suited checklist. *"The checklist must describe exactly the intended operation" *(A) and *"The checklist, and its content, must directly reflect the specific operation and its surroundings" *(E).

### Human factors during checklist utilisation

*"The checklist must foster resilient communication" *(G). The informants underlined the importance of understanding and communication to minimize the risk of misunderstandings and to optimize flow during checklist use (Table 4).

**Table 4:** Communication and understanding during checklist performance.

Context	*"The end user must be familiar with the background of each point in the checklist" *(C)
Terminology and communication	*"It is important with a predefined and agreed upon phraseology - closed loop communication - " *(F)
	
	*"The language must be adapted to the present terminology within the organisation " *(H)

Understanding and situational awareness	*"The understanding of the text must be unambiguous or clearly defined" *(H)
	
	*"Be aware of automation, i.e. performing the checklist without being mentally present" *(A)

### Revisions and validation

All the informants described a thorough system for checklist revisions. One informant (E) said that revisions are important for two reasons; firstly, *"To maintain a dynamic and up-to-date checklist" *and secondly, *"To build a culture where the sharp-end feels that their feedback is valuable to the organization"*. All informants used the terms "revision" and "validation" imprecisely and interchangeably. The key "validation" measure described by the informants was the absence of serious accidents and adverse events after introducing the checklists. Table 5 lists the different methods and approaches for validation and revision.

**Table 5:** Methods and approaches for checklist revision and validation

Simulation	*"Simulation is a good method for validation and testing the usefulness and feasibility of checklists" *(C)
	
	*"Use simulation to practise checklist utilisation" *(E)
	
	"*Checklists should be validated regularly according to a plan, in a simulator*" (G)
AE analysis	*"Use the experience from adverse events during debrief to validate and revise checklists" *(E)

Feed-back from end-users	*"The validating and revision group must listen to the end-users' experiences and frustrations" *(D)
	
	*"Revision could be done by feedback from the end-user. It is important that the end-users understand that their experiences are wanted and expected, and that such feed-back may have consequences." *(B)

## Discussion

Experiences with checklists in health care are limited compared with HROs. Few studies have explored these organisations' acquired knowledge in checklist development and implementation. Our findings suggest several issues worth considering when introducing medical checklists.

### Translating ideas from HROs to patient safety

HROs undoubtedly have thorough and trustworthy experience with checklists, but some have questioned whether health care has gone too far in translating ideas from HROs in general [24]. HROs and medicine have many similarities concerning human factors and complex operations. They both fall prey to the limitations of human pathophysiology and the many challenges of man-machine interface. This study is limited to experiences with checklists and will not discuss the many additional issues concerned with building a safety culture.

### Acceptance of checklists

Implementing checklists involves many conflicting interests, including the organisational culture and workflow, which are often affected [25]. Oversimplification of potential challenges could easily lead to conflicts between fractions of adopters and opponents. There are several reports of low compliance when checklists are introduced in health care [10,26]. Our data highlights some important assumptions about achieving checklist acceptance. Firstly, there must be an identified need or problem for which a checklist is considered the right solution. A process is doomed to fail if the safety manager or department head try to force the "sharp-end" personnel to use a checklist to solve an issue that is not recognised as a problem. Secondly, be aware of challenging physicians' self-esteem and historically strong cultural autonomy. Human beings (including physicians) have cognitive limitations such as difficulty in carefully attending to several things simultaneously [21]. An effective checklist will enhance performance during high workload and stressful conditions by freeing mental capacity to perform important tasks in the correct manner and order [27]. However, checklists should not deprive the operator of the opportunity to use common sense or independently make more reasonable decisions.

### Proximity and ownership

The participants in our study emphasized that all stakeholders must have ownership, not only for the final checklist but for the entire development process. These views are consistent with the encouragement to make local modifications to the WHO Surgical Safety Checklist [28]. Recent critical reports from the UK call for an urgent nationwide checklist revision [29,30]. These challenges regarding checklist acceptance may be the result of failed checklist implementation locally, and lack of proximity between the checklist-owner and end-users.

### Checklist lay-out and design

The visual elements of a checklist are important and will directly influence its efficacy and feasibility [14]. There are several guidelines for checklist lay-out and design from both medicine and aviation [31-33]. All informants emphasized the importance of short checklists. Long lists should be divided into shorter sections or separate lists. One of the informants from aviation (E) described how they had divided the long "normal procedures checklist" into three chronological sections; "before take off", "in-flight" and "before landing". The WHO safe surgery checklist follows the same logic ("check-in", "time-out", and "sign-out"). Checklists must be easy to find and use, easy to understand, and short.

### Simulator training

All but one of the informants had regular and mandatory checklist-training in full scale simulators. Such simulation is an expensive and time consuming training method, but after decades of experience this is still the preferred method (gold standard) for team and checklist training in these HROs. We also believe that team training is the key for successful checklist implementation in health care. Local team training with low cost and simple technical fidelity is a feasible method for rehearsing complex operations [34]. Success in checklist implementation is probably more a matter of will, enthusiasm, and organisational competence than of available time and financial resources [35].

### Flow of expertise and delegation of authority

Health care has traditionally had a strong hierarchical structure. Such a culture can discourage information exchange, resulting in anxiety or unwillingness to provide feedback on a checklist [36]. HROs cultivate a diversity of expertise; hence, authority migrates to the personnel with process-specific knowledge, independent of their hierarchical organisational position [17]. All the informants described a system where the sharp-ends' experiences are regarded as a valuable and essential part of the revision process. Resilience is a key issue in this context, and has been defined as "the intrinsic ability of a system to adjust its functioning prior to, during, or following changes and disturbances."[37] One cornerstone of resilience is to understand and obtain experiences from both the sharp and blunt end [38]. A self-reinforcing and destructive process may develop if a hierarchical structure prevents feedback from important stakeholders during checklist implementation.

### Checklists reduce prospective memory failures

Prospective memory (PM) describes the ability to remember to carry out actions (in the near future) that are planned after a delay or interruption [39]. Unpredictability, delays, and interruptions are frequent in health care [40]. During a stressful situation, memory is also likely to be more error-prone [41]. Cognitive aids like checklists are shown to increase performance in health care when solving complex and time-critical tasks [42,43]. All the informants in our study described a culture where cognitive aids had an essential role. Medicine has traditionally relied on memory as the basis of diagnosis and treatment [42]. It has been argued that "PM failures in medicine must not be forgotten any more" [39].

### Strengths and limitations

The key strength of this study is the diversity of the informants, who provided a broad information source. Despite the nature of their often classified operations, none of those invited to take part in the study declined to participate. This enabled insight from an often inaccessible safety culture. Additionally, each informant had 10-30 years of cultural experience and an understanding of their organisations. Still, the informants' experiences may not have reflected all the relevant issues regarding checklist development and implementation from their own HRO, and the lack of female informants may also have limited the data to some degree.

### Further research

In this study we have obtained experiences from informants representing six different HROs. It is likely to believe that these informants, together in an expert consensus process, could have developed and provided further understanding in checklist implementation.

Checklists in HROs are highly valued and given resources to develop, use and validate. Critical comments in health care are concerned if checklists may be cost-ineffective [44]. This line of criticism is debated claiming that checklists are a cost saving quality improvement strategy [45]. Studies concerning cost - benefit should be encouraged.

## Conclusion

Checklists are one of the cornerstones of the safety culture of HROs, and their main purpose is to free workers' mental capacity to fully attend to operations. Close cooperation with the "sharp-end" and ownership for all stakeholders throughout the entire checklist implementation process are fundamental requirements in HROs. Despite complex and hazardous operations, HROs manage to keep checklists short. Checklists must reflect the processes they are intended to structure. Simulation is an essential method for training in reliable communication, increasing checklist use, and identifying the need for and implementing revisions.

### What is already known on this topic

• The introduction of checklists into health care has been slow, despite documented effects on morbidity and mortality.

• HROs often use checklists, and aviation has been described as the archetype of HROs with extensive checklist experience.

• Checklists can be viewed as simple tools, but their implementation is not so simple.

### What this study adds

• A recognised and predefined problem is a requirement for checklist acceptance.

• The end-users ("sharp end" personnel) are the key stakeholders throughout the checklist development process.

• Proximity for all stakeholders throughout the entire checklist implementation process is fundamental.

• Even for complex and hazardous operations, HROs have managed to keep checklists short.

• Simulation is a suitable and necessary part of checklist testing, implementation, and revision

## Abbreviations

AE: Adverse Event; HRO: High reliability organisations; WHO: World Health Organisation.

## Competing interests

The authors declare that they have no competing interests.

## Authors' contributions

OT and GB conceived and designed the study. OT made all the interviews and led the analysis and writing process. AE made an extensive contribution in method design. ES, HML and JKH took part in the Delphi approach. All authors contributed to the writing of the final version of the paper, and approved the final manuscript. OT is the guarantor.
